# Drug-target interaction prediction via class imbalance-aware ensemble learning

**DOI:** 10.1186/s12859-016-1377-y

**Published:** 2016-12-22

**Authors:** Ali Ezzat, Min Wu, Xiao-Li Li, Chee-Keong Kwoh

**Affiliations:** 1 School of Computer Science & Engineering, Nanyang Technological University, Nanyang Ave., Singapore, 639798 Singapore; 2 Institute for Infocomm Research (I2R), A*Star, Fusionopolis Way, Singapore, 138632 Singapore

**Keywords:** Drug-target interaction prediction, Class imbalance, Between-class imbalance, Within-class imbalance, Small disjuncts, Ensemble learning

## Abstract

**Background:**

Multiple computational methods for predicting drug-target interactions have been developed to facilitate the drug discovery process. These methods use available data on known drug-target interactions to train classifiers with the purpose of predicting new undiscovered interactions. However, a key challenge regarding this data that has not yet been addressed by these methods, namely *class imbalance*, is potentially degrading the prediction performance. Class imbalance can be divided into two sub-problems. Firstly, the number of known interacting drug-target pairs is much smaller than that of non-interacting drug-target pairs. This imbalance ratio between interacting and non-interacting drug-target pairs is referred to as the *between-class* imbalance. Between-class imbalance degrades prediction performance due to the bias in prediction results towards the majority class (i.e. the non-interacting pairs), leading to more prediction errors in the minority class (i.e. the interacting pairs). Secondly, there are multiple types of drug-target interactions in the data with some types having relatively fewer members (or are less represented) than others. This variation in representation of the different interaction types leads to another kind of imbalance referred to as the *within-class* imbalance. In within-class imbalance, prediction results are biased towards the better represented interaction types, leading to more prediction errors in the less represented interaction types.

**Results:**

We propose an ensemble learning method that incorporates techniques to address the issues of between-class imbalance and within-class imbalance. Experiments show that the proposed method improves results over 4 state-of-the-art methods. In addition, we simulated cases for *new* drugs and targets to see how our method would perform in predicting their interactions. New drugs and targets are those for which no prior interactions are known. Our method displayed satisfactory prediction performance and was able to predict many of the interactions successfully.

**Conclusions:**

Our proposed method has improved the prediction performance over the existing work, thus proving the importance of addressing problems pertaining to class imbalance in the data.

**Electronic supplementary material:**

The online version of this article (doi:10.1186/s12859-016-1377-y) contains supplementary material, which is available to authorized users.

## Background

On average, it takes over a dozen years and around 1.8 billion dollars to develop a drug [1]. Moreover, most of the drugs being developed fail to reach the market due to reasons pertaining to toxicity or low efficacy [2]. To mitigate the risks and costs inherent in traditional drug discovery, many pharmaceutical companies resort to *drug repurposing* or *repositioning* where drugs already on the market may be reused for novel disease treatments that differ from their original objective and purpose [3].

Intuitively, repurposing a known drug to treat new diseases is convenient and cost-effective for the following two reasons. Firstly, since the drug being repurposed is one that is already on the market (i.e. already approved by the FDA), this implicitly means that it already passed clinical trials that ensure the drug is safe to use. Secondly, the drug being repurposed has already been studied extensively, so many of the drug’s properties (e.g. interaction profile, therapeutic or side effects, etc.) are known before initiating the drug repurposing effort. As such, drug repurposing helps facilitate and accelerate the research and development process in the drug discovery pipeline [2].

Many data sources are publicly available online that support efforts in computational drug repositioning [4]. Based on the types of data being used, different methods and procedures have been proposed to achieve drug repositioning [5]. In this paper, we particularly focus on *global-scale drug-target interaction prediction*; that is, leveraging information on known drug-target interactions, we aim to predict or prioritize new *previously unknown* drug-target interactions to be further investigated and confirmed via experimental wet-lab methods later on.

The main benefit of this technique for drug repositioning efforts is that, given a protein of interest (e.g. its gene is associated with a certain disease), many FDA-approved drugs may simultaneously be computationally screened to determine good candidates for binding [6]. As previously mentioned, using an approved drug as a starting point in drug development has desirable benefits regarding cost, time and effort spent in developing the drug. In addition, other benefits of this technique include the screening of potential off-targets that may cause undesired side effects, thus facilitating the detection of potential problems early in the drug development process. Finally, new predicted targets for a drug could improve our understanding of its actions and properties [7].

Efforts involving global-scale prediction of drug-target interactions have been fueled by the availability of publicly available online databases that store information on drugs and their interacting targets, such as KEGG [8], DrugBank [9], ChEMBL [10] and STITCH [11].

These efforts can be divided into three categories. The first category is that of *ligand-based* methods where the drug-target interactions are predicted based on the similarity between the target proteins’ ligands. A problem with this category of methods is that many target proteins have little or no ligand information available, which limits the applicability of these methods [12].

*Docking simulation* methods represent the second category of approaches for predicting drug-target interactions. Although they have been successfully used to predict drug-target interactions [13, 14], a limitation with these methods is that they require the 3D structures of the proteins, which is a problem because not all proteins have their 3D structures available. In fact, most membrane proteins (which are popular drug targets) do not have resolved 3D structures, as determining their structures is a challenging task [15].

The third category is the *chemogenomic* approaches which simultaneously utilize both the drug and target information to perform predictions. Chemogenomic methods come in a variety of forms. Some are kernel-based methods that make use of information encoded in both drug and target similarity matrices to perform predictions [16–21], while other chemogenomic methods use graph-based techniques, such as random walk [22] and network diffusion [23].

In this paper, we focus on a particular type of chemogenomic methods, namely *feature-based* methods, where drugs and targets are represented with sets of descriptors (i.e. feature vectors). For example, He et al. represented drugs and targets using common chemical functional groups and pseudo amino acid composition, respectively [24], while Yu et al. used molecular descriptors that were calculated using the DRAGON package [25] and the PROFEAT web server [26] for drugs and targets, respectively [27]. Other descriptors have also been used such as position-specific scoring matrices [28], 2D molecular fingerprints [29], MACCS fingerprints [30], and domain and PubChem fingerprints [31].

In general, many of the existing methods treat drug-target interaction prediction as a binary classification problem where the *positive* class consists of interacting drug-target pairs and the *negative* class consists of non-interacting drug-target pairs. Clearly, there exists a *between-class* (or inter-class) imbalance as the number of the non-interacting drug-target pairs (or majority negative class instances) far exceeds that of the interacting drug-target pairs (or minority positive class instances). This results in biasing the existing prediction methods towards classifying instances into the majority class to minimize the classification errors [32]. Unfortunately, minority class instances are the ones of interest to us. A common solution that was used in previous studies (e.g. [27]) is to perform random sampling from the majority class until the number of sampled majority class instances matches that of the minority class instances. While this considerably mitigates the bias problem, it inevitably leads to the discarding of useful information (from the majority class) whose inclusion may lead to better predictions.

The other kind of class imbalance that also degrades prediction performance, but has not been previously addressed, is the *within-class* (or intra-class) imbalance which takes place when *rare cases* are present in the data [33]. In our case, there are multiple different types of drug-target interactions in the positive class, but some of them are represented by relatively fewer members than others and can be considered as less well-represented interaction groups (also known as *small concepts* or *small disjuncts*). If not processed well, they are a source of errors because predictions would be biased towards the well-represented interaction types in the data and ignore these specific small concepts.

In this paper, we propose a simple method that addresses the two imbalance problems stated above. Firstly, we provide a solution for the high imbalance ratio between the minority and majority classes while greatly decreasing the amount of information discarded from the majority class. Secondly, our method also deals with the within-class imbalance prevalent in the data by balancing the ratios between the different concepts inside the minority class. Particularly, we first perform clustering to detect homogenous groups where each group corresponds to one specific concept and the interactions within smaller groups are relatively easier to be incorrectly classified. As such, we artificially enhance small groups via oversampling, which essentially helps our classification model focus on these small concepts to minimize classification errors.

## Data

This section provides our dataset information including raw drug-target interaction data and the data representation that turns each drug-target pair into its feature vector representation.

### Drug-target interaction data

The interaction data used in this study was collected recently from the DrugBank database [9] (version 4.3, released on 17 Nov. 2015). Some statistics regarding the collected interaction data are given in Table 1. In total, there are 12674 drug-target interactions between 5877 drugs and their 3348 protein interaction partners. The full lists of drugs and targets used in this study as well as the interaction data (i.e. which drugs interact with which targets) have been included as supplementary material [see Additional files 1, 2 and 3].

**Table 1:** Statistics of the interaction dataset used in this study

Drugs	Targets	Interactions
5877	3348	12674

### Data representation

After having obtained the interaction data, we generated features for the drugs and targets respectively. Particularly, descriptors for drugs were calculated using the *Rcpi* [34] package. Examples of drug features include constitutional, topological and geometrical descriptors among other molecular properties. Note that biotech drugs have been excluded from this study as Rcpi could only generate such features for small-molecule drugs. The statistics given in Table 1 reflect our final dataset after the removal of these biotech drugs.

Now, we describe how target features were obtained. Since it is generally assumed that the complete information of a target protein is encoded in its sequence [24], it may be intuitive to represent targets by their sequences. However, representing the targets this way is not suitable for machine learning algorithms because the length of the sequence varies from one protein to another. To deal with this issue, an alternative to using the raw protein sequences is to compute (from these same sequences) a number of different descriptors corresponding to various protein properties. The list of computed features is intended to be as comprehensive as possible so that it may, as much as possible, convey all the information available in the genomic sequences that they were computed from. Computing this list of features for each of the targets lets them be represented using fixed-length feature vectors that can be used as input to machine learning methods. In our work, the target features were computed from their genomic sequences with the help of the *PROFEAT* [26] web server.

The features that have been used to represent targets in this work are descriptors related to amino acid composition; dipeptide composition; autocorrelation; composition, transition and distribution; quasi-sequence-order; amphiphilic pseudo-amino acid composition and total amino acid properties. Note that a similar list of features was used previously in [27]. Subsets of these features have also been used in other previous studies concerning drug-target interaction prediction [24, 35]. More information regarding the computed features can be accessed at the online documentation webpage of the PROFEAT web server where all the features are described in detail.

After generating features for drugs and targets, there were features that had constant values among all drugs (or targets). Such features were removed as they would not contribute to the prediction of drug-target interactions. Furthermore, there were other features that had missing values for some of the drugs (or targets). For each of these features, the missing values were replaced by the mean of the feature over all drugs (or targets). In the end, 193 and 1290 features remained for drugs and targets, respectively. The full lists of drug features and target features used in this study have been included as supplementary material [see Additional files 4 and 5].

Next, every drug-target pair is represented by feature vectors that are formed by concatenating the feature vectors of the corresponding drug and target involved. For example, a drug-target pair (*d,t*) is represented by the feature vector,

$$\quad\quad\quad [d_{1}, d_{2}, \ldots, d_{193}, t_{1}, t_{2}, \ldots, t_{1290}], $$

where [*d*_1_,*d*_2_,…,*d*_193_] is the feature vector corresponding to drug *d*, and [*t*_1_,*t*_2_,…,*t*_1290_] is the feature vector corresponding to target *t*. Hereafter, we also refer to these drug-target pairs as *instances*. Finally, to avoid potential feature bias in its original feature values, all features were normalized to the range [0,1] using min-max normalization before performing drug-target interaction prediction as follows

$$\begin{array}{@{}rcl@{}}  \forall i=1,\ldots,193 &,& d_{i}=\frac{d_{i}-min(d_{i})}{max(d_{i})-min(d_{i})} \\  \forall j=1,\ldots,1290 &,& t_{j}=\frac{t_{j}-min(t_{j})}{max(t_{j})-min(t_{j})}. \end{array} $$

The feature vectors that were computed for the drugs and targets have been included as supplementary material [see Additional files 6 and 7].

## Methods

The proposed method was developed with an intention to deal with two key imbalance issues, namely the between-class imbalance and the within-class imbalance. Here, we describe in detail how each of these imbalance issues was handled. For notation, we use *P* to refer to the set of *positive* instances (i.e. the *known* experimentally verified drug-target interactions) and use *N* to refer to the remaining *negative* instances (consisting of all other drug-target pairs that do not occur in *P*).

Technically speaking, these remaining instances should be called *unlabeled* instances as they have not been experimentally verified to be true non-interactions. In fact, we believe that some of the instances in *N* are actually true drug-target interactions that have not been discovered yet. Nevertheless, to simplify our discussion, we refer to them as negative instances since we assume the proportion of non-interactions in *N* to be quite high.

### Our proposed algorithm

We propose a simple ensemble learning method where the prediction results of the different base learners are aggregated to produce the final prediction scores. For base learners, our ensemble method uses decision trees which are popularly used in ensemble methods (e.g. random forest [36]). Decision trees are known to be *unstable learners*, meaning that their prediction results are easily perturbed by modifying the training set, making them a good fit with ensemble methods which make use of the *diversity* in their base learners to improve prediction performance [37].

It is generally known that an ensemble learning method improves prediction performance over any of its constituent base learners only if they are uncorrelated. Intuitively, if the base learners of an ensemble method were identical, then there would no gain in prediction performance at all. As such, adding diversity to the base learners is important.

One way of introducing diversity to the base learners that is used in our method is supplying each base learner with a different training set. Another way of adding diversity that we also employ here is *feature subspacing*; that is, for each of the base learners, we represent the instances using a different subset of the features. More precisely, for each base learner, we randomly select two thirds of the features to represent the instances.

Algorithm 1 shows our pseudocode for the overall architecture of our proposed method where the specific steps for handling the two imbalance issues are discussed in the following subsections. Following is a summary of the method:

- *T* decision trees are trained (*T* is a parameter),
- Prediction results of the *T* trees are aggregated by simple averaging to give the final prediction scores.
- For each decision tree, *tree*_*i*_:

1. Randomly select a subset of the features, *F*_*i*_.
2. Obtain *P*_*i*_ by performing feature subspacing on *P* using *F*_*i*_.
3. Oversample *P*_*i*_.
4. Randomly sample *N*_*i*_ from *N* such that |*N*_*i*_|=|*P*_*i*_|.
5. Remove instances of *N*_*i*_ from *N*.
6. Modify *N*_*i*_ by performing feature subspacing on it using *F*_*i*_.
7. Train *tree*_*i*_ using the positive set *P*_*i*_ and the negative set *N*_*i*_ as the training set.

### Within-class imbalance

We are now ready to explain the *OVERSAMPLE*(*P*_*i*_) in Algorithm 1. As mentioned in the introduction section, within-class imbalance refers to the presence of specific types of interactions in the positive set *P* that are under-represented in the data as compared to other interaction types. Such cases are referred to as *small concepts*, and they are a source of errors because prediction algorithms are typically biased in that they favor the better represented interaction types in the data so as to achieve better generalization performance on unseen data [33].

To deal with this issue, we use the *K*-means++ clustering method [38] to cluster the data into *K* homogenous clusters (*K* is a parameter) where each cluster corresponds to one specific concept. This results in interaction groups/clusters of different sizes. The assumption here is that the small clusters (i.e. those that contain few members) correspond to the rare concepts (or small disjuncts) that we are concerned about. Supposing that the size of the biggest cluster is *maxClusterSize*, all clusters are re-sampled until their sizes are equal to *maxClusterSize*. This way, all concepts become represented by the same number of members and are consequently treated equally in training our classifier. Essentially, this is similar in spirit to the idea of boosting [39] where examples that are incorrectly classified have their weights increased so that classification methods will focus on the hard-to-classify examples to minimize the classification errors.

Algorithm 2 shows the pseudocode for the oversampling procedure. *P*_*i*_ is first clustered into *K* clusters of different sizes. After determining the size of the biggest of these clusters, *maxClusterSize*, all clusters are re-sampled until their sizes are equal to *maxClusterSize*. The re-sampled clusters are then assigned to *P*_*i*_ before returning it to the main algorithm in the “Our proposed algorithm” subsection.



An issue that we considered while implementing the oversampling procedure was that of data noise. Indeed, emphasizing small concept data can become a counter-productive strategy if there is much noise in the data. However, the data used in this study was obtained from DrugBank [9], and since the data stored there is regularly curated by experts, we have high confidence in the interactions observed in our dataset. In other words, the interactions (or positive instances) are quite reliable and are expected to contain little to no noise. On the other hand, the negative instances are expected to contain noise since, as mentioned earlier, these negative instances are actually unlabeled instances that likely contain interactions that have not been discovered yet. Here, we only amplify the importance of small-concept data from the positive set (i.e. the set of known drug-target interactions). Since the positive instances being emphasized are highly reliable, the potential impact of noise on the prediction performance is minimal.

### Between-class imbalance

Between-class imbalance refers to the bias in the prediction results towards the majority class, leading to errors where minority examples are classified into the majority class. We wanted to ensure that predictions are not biased towards the majority class while, at the same time, decrease the amount of useful majority class information being discarded. To that end, a different set of negative instances *N*_*i*_ is randomly sampled from *N* for each base learner *i* such that |*N*_*i*_|=|*P*_*i*_|. The 1:1 ratio of the sizes of *P*_*i*_ and *N*_*i*_ eliminates the bias of the prediction results towards the majority class. Moreover, whenever a set of negative instances *N*_*i*_ is formed for a base learner, its instances are excluded from consideration when we perform random sampling from *N* for future base learners. The different non-overlapping negative sets that are formed for the base learners lead to better coverage of the majority class in training the ensemble classifier.

Note that, to improve coverage of the majority class in training, the value of the parameter *T* needs to be increased where *T* is the number of base learners in the ensemble method, which also determines the number of the times that we want to draw instances from the negative set *N*. In general, with the increase of the value of *T*, more useful information from the majority class will be incorporated to build our final classification model.

## Results and discussion

In this section, we have performed comprehensive experiments in which we compare our proposed technique with 4 existing methods. Below, we first elaborate on our experimental settings. Next, we provide details of our cross-validation experiments and comparison results. Finally, we focus on predicting interactions for new drugs and new targets, which is crucial for both novel drug design and drug repositioning tasks.

### Experimental settings

To evaluate our proposed method, we conducted an empirical comparison with 2 state-of-the-art methods and 2 baseline methods. Particularly, *Random Forest* and *SVM* are existing state-of-the-art methods that were both used in a recent work for predicting drug-target interactions [27]. Note that the parameters for these 2 methods were set to the default optimal values supplied in [27]. We also included two baseline methods, namely *Decision Tree* and *Nearest Neighbor*. For Decision Tree, we employed the *fitctree* built-in package in MATLAB and used the default parameter values as they were found to produce reasonable good results. As for Nearest Neighbor, it produces a prediction score for every test instance *a* by computing its similarity to the nearest neighbor *b* from the minority class *P* (which contains the known interacting drug-target pairs) based on the following equations,

$$score_{a}=max_{b}(sim(a,b)),\quad b\in P $$

$$sim(a,b)=exp\left(-\frac{||a-b||^{2}}{|F|}\right), $$

where |*F*| is the number of features.

For the above 4 competing methods, they all used *P* as the positive set, while the negative set was sampled randomly from *N* until its size reached |*P*|. In contrast, our method oversampled *P* for each base learner *i*, giving *P*_*i*_, and a negative set *N*_*i*_ was sampled from *N* for each base learner *i* such that |*N*_*i*_|=|*P*_*i*_|. Note that different base learners have used different negative sets in our proposed method. In addition, the parameters *K* and *T* for our method were set to 100 and 500, respectively, to generate sufficient homogenous clusters and leverage more negative data.

### Cross validation experiments

To study the prediction performance of our proposed method, we performed a standard 5-fold cross validation and computed the AUC for each method (i.e. the area under the ROC curve). More precisely, for each of the methods being compared, 5 AUC scores were computed (one for each fold) and then averaged to give the final overall AUC score. Note that AUC is known to be insensitive to skewed class distributions [40]. Considering that the drug target interaction dataset used in this study is highly imbalanced (we have much more negatives than positives), AUC score is thus a suitable metric for evaluation of the different computational methods.

**Fig. 1:**
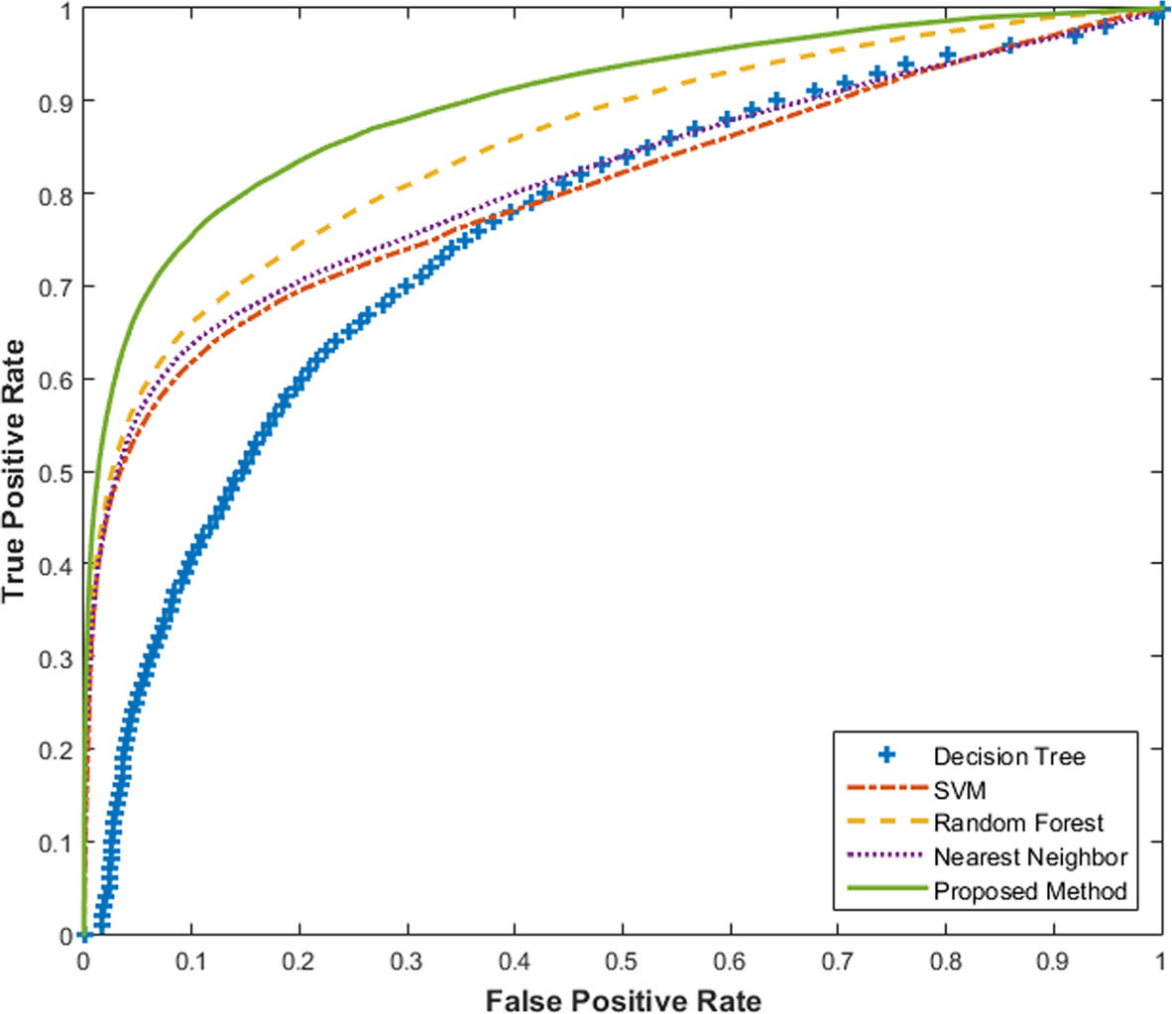
Plot of ROC *curves* of the different methods. ROC curves for the different methods are plotted together, providing a visual comparison between their prediction performances

Figure 1 shows the ROC curves for various methods. It is obvious that the ROC curve for our proposed method dominates those for the other methods, implying that it has a higher AUC score. In particular, Table 2 shows the AUC scores for different methods in details. Our proposed method achieves an AUC of 0.900 and performs significantly better than other existing methods.

**Table 2:** AUC Results of cross validation experiments

Decision Tree	0.760 (0.004)
SVM	0.804 (0.004)
Nearest Neighbor	0.814 (0.003)
Random Forest	0.855 (0.006)
**Proposed Method**	**0.900 (0.006)**

Standard deviations are included between parentheses. Best AUC is indicated in bold

As shown in Table 2, the second best method is Random Forest. Moreover, our method is similar to Random Forest in that they are both ensembles of decision trees with feature subspacing. Both our proposed method and Random Forest perform very well in drug-target interaction prediction, showing that ensemble methods are indeed superior to achieve good prediction performance. However, our method differs from Random Forest in two perspectives. Firstly, Random Forest performs bagging on a single sampled negative set for each base learner, while our method leverages multiple non-overlapping negative sets for different base learners. Secondly, our method also oversamples the positive set in a way that is intended to deal with the within-class imbalance, while Random Forest does not. Due to these 2 differences, our method achieved an AUC of 0.900, which is 4.5% higher than Random Forest with an AUC of 0.855. This supports our claim that dealing with class imbalance in the data is important for improving the prediction performance.

### Predicting interactions for new drugs and targets

A scenario that may occur in drug discovery is that we may have a target protein of interest for which no information on interacting drugs is available. This is typically a more challenging case than if we had information on drugs that the target protein is already known to interact with. A similar scenario that occurs frequently in practice is that we have new compounds (potential drugs) for which no interactions are known yet, and we want to determine candidate target proteins that they may interact with. When there is no interaction information on a drug or target, they are referred to as a *new drug* or a *new target*.

To test the ability of our method to correctly predict interactions in these challenging cases, we simulated the cases of new drugs and targets by leaving them out of our dataset, training with the rest of the data and then obtaining predictions for these new drugs and new targets. In our case studies, we ranked the predicted interactions and investigated the top 20 interactions. In particular, we investigated two drugs, *Aripiprazole* and *Theophylline*, and two targets, *Glutamate receptor ionotropic, kainate 2* and *Xylose isomerase*, respectively. Tables 3 and 4 show the top 20 predictions for these drugs and targets.

**Table 3:** Top 20 targets predicted for *Aripiprazole* and *Theophylline*

Aripiprazole		Theophylline	
Rank	Target	Rank	Target
**1**	**5-hydroxytryptamine receptor 2A**	**1**	**cAMP-specific 3’,5’-cyclic phosphodiesterase 4A**
**2**	**Alpha-1B adrenergic receptor**	**2**	**Histone deacetylase 2**
**3**	**Muscarinic acetylcholine receptor M2**	**3**	**Adenosine receptor A2a**
**4**	**5-hydroxytryptamine receptor 2C**	**4**	**Adenosine receptor A1**
**5**	**D(1) dopamine receptor**	**5**	**cGMP-inhibited 3’,5’-cyclic phosphodiesterase A**
**6**	**Alpha-2C adrenergic receptor**	**6**	**cAMP-specific 3’,5’-cyclic phosphodiesterase 4B**
**7**	**Histamine H1 receptor**	**7**	**Adenosine receptor A2b**
**8**	**Muscarinic acetylcholine receptor M3**	**8**	**cGMP-specific 3’,5’-cyclic phosphodiesterase**
**9**	**D(2) dopamine receptor**	9	Adenosine receptor A3
**10**	**Muscarinic acetylcholine receptor M1**	10	Thymidylate synthase
**11**	**5-hydroxytryptamine receptor 1B**	11	Histone deacetylase 1
12	Delta-type opioid receptor	12	Cyclin-dependent kinase 2
**13**	**D(4) dopamine receptor**	13	Reverse transcriptase/RNaseH
**14**	**D(3) dopamine receptor**	14	Cap-specific mRNA (nucleoside-2’-O-)-methyltransferase
**15**	**5-hydroxytryptamine receptor 1D**	15	Multi-sensor signal transduction histidine kinase
**16**	**Alpha-1 adrenergic receptor**	16	Alpha-1 adrenergic receptor
**17**	**Muscarinic acetylcholine receptor M5**	17	Serine/threonine-protein kinase pim-1
**18**	**Muscarinic acetylcholine receptor M4**	18	Serine-protein kinase ATM
**19**	**Alpha-2B adrenergic receptor**	19	Proto-oncogene tyrosine-protein kinase Src
**20**	**5-hydroxytryptamine receptor 1A**	20	Phosphatidylinositol 4,5-bisphosphate 3-kinase
			catalytic subunit alpha isoform

Targets in bold are the true known targets of the drugs

**Table 4:** Top 20 drugs predicted for *Glutamate receptor ionotropic, kainate 2* and *Xylose isomerase*

Glutamate receptor ionotropic, kainate 2		Xylose isomerase	
Rank	Drug	Rank	Drug
**1**	**Metharbital**	**1**	**D-Xylitol**
**2**	**Butabarbital**	2	alpha-D-Xylopyranose
**3**	**Pentobarbital**	**3**	**L-Xylopyranose**
**4**	**Thiopental**	4	beta-D-Ribopyranose
**5**	**Butethal**	**5**	**D-Sorbitol**
**6**	**Secobarbital**	**6**	**D-Xylulose**
**7**	**Talbutal**	**7**	**Vitamin C**
**8**	**Hexobarbital**	**8**	**2-Methylpentane-1,2,4-Triol**
**9**	**Barbital**	9	Tris-Hydroxymethyl-Methyl-Ammonium
**10**	**Amobarbital**	**10**	**(4r)-2-Methylpentane-2,4-Diol**
**11**	**Phenobarbital**	11	Ethanol
**12**	**Butalbital**	12	Beta-D-Glucose
**13**	**Aprobarbital**	13	D-Allopyranose
**14**	**Methylphenobarbital**	14	2-Deoxy-Beta-D-Galactose
**15**	**Primidone**	15	Tris
16	Lysine Nz-Carboxylic Acid	16	3-O-Methylfructose in Linear Form
**17**	**Domoic Acid**	17	Dithioerythritol
**18**	**Heptabarbital**	18	(2s,3s)-1,4-Dimercaptobutane-2,3-Diol
19	Vitamin A	19	1,4-Dithiothreitol
20	Mephenytoin	20	Glycerol

Drugs in bold are true known drugs of the targets

In our dataset, *Aripiprazole* and *Theophylline* are known to interact with 25 and 8 targets, respectively. Out of the top 20 predicted targets for *Aripiprazole*, 19 were correctly predicted as shown in Table 3. For *Theophylline*, all of its 8 interactions were highly ranked in its top 20 list.

Moreover, *Glutamate receptor ionotropic, kainate 2* and *Xylose isomerase* have 20 and 7 interacting drugs in our dataset. Out of the top 20 predicted drugs for *Glutamate receptor ionotropic, kainate 2*, 17 were successfully predicted as shown in Table 4. For *Xylose isomerase*, all its 7 drugs were predicted in the top 20. These promising results show that our method is indeed reliable for predicting interactions in the cases of *new* drugs or targets.

Finally, we investigated the possibility that some of the unconfirmed interactions in Tables 3 and 4 might be true. For example, we observed that *Delta-type opioid receptor* is indeed a target for *Aripiprazole*, which was confirmed from the T3DB online database [41]. We have also confirmed, using the STITCH online database [11], that *Adenosine receptor A3* and *Histone deacetylase 1* are true targets of *Theophylline* as well. These findings suggest that the unconfirmed interactions in Tables 3 and 4 may be true interactions that have not been discovered yet.

## Conclusion

We proposed a simple yet effective ensemble method for predicting drug-target interactions. This method includes techniques for dealing with two types of class imbalance in the data, namely between-class imbalance and within-class imbalance. In our experiments, our method has demonstrated significantly better prediction performance than that of the state-of-the-art methods via cross-validation. In addition, we simulated new drug and new target prediction cases to evaluate our method’s performance under such challenging scenarios. Our experimental results show that our proposed method was able to highly rank true known interactions, indicating that it is reliable in predicting interactions for new compounds or previously untargeted proteins. This is particularly important in practice for both identifying new drugs and detecting new targets for drug repositioning.

## Additional files

Additional file 1 Drug IDs. This file contains the DrugBank IDs of the drugs used in this study. (46 kb TXT)

Additional file 2 Target IDs. This file contains the UniProt IDs of the targets used in this study. (23 kb TXT)

Additional file 3 Drug-target interaction matrix. This file contains the known drug-target interactions in the form of a matrix, where rows represent the drugs, and the columns represent the targets. Drug-target pairs that interact have a 1 in their corresponding cell and 0 otherwise. (37500 kb TXT)

Additional file 4 List of drug features. This file contains the names of the drug features used in this study. More details on the features can be found at: http://bioconductor.org/packages/release/bioc/html/Rcpi.html (1 kb TXT)

Additional file 5 List of target features. This file contains the names of the target features used in this study. More details on the features can be found at: http://bidd2.nus.edu.sg/prof/manual/prof.htm (16 kb TXT)

Additional file 6 Drug feature vectors. This file contains the feature vectors for the drugs. (6180 kb TXT)

Additional file 7 Target feature vectors. This file contains the feature vectors for the targets. (24400 kb TXT)

## References

[1] Paul SM, Mytelka DS, Dunwiddie CT, Persinger CC, Munos BH, Lindborg SR, Schacht AL (2010). How to improve r&d productivity: the pharmaceutical industry’s grand challenge. Nat Rev Drug Discov.

[2] Novac N (2013). Challenges and opportunities of drug repositioning. Trends Pharmacol Sci.

[3] Ashburn TT, Thor KB (2004). Drug repositioning: identifying and developing new uses for existing drugs. Nat Rev Drug Discov.

[4] Li J, Zheng S, Chen B, Butte AJ, Swamidass SJ, Lu Z (2016). A survey of current trends in computational drug repositioning. Brief Bioinformatics.

[5] Jin G, Wong STC (2014). Toward better drug repositioning: prioritizing and integrating existing methods into efficient pipelines. Drug Discov Today.

[6] Xie L, Kinnings SL, Xie L, Bourne PE. Drug repositioning: Bringing new life to shelved assets and existing drugs. John Wiley & Sons, Inc. 2012. doi:10.1002/9781118274408.

[7] Keiser MJ, Setola V, Irwin JJ, Laggner C, Abbas AI, Hufeisen SJ, Jensen NH, Kuijer MB, Matos RC, Tran TB (2009). Predicting new molecular targets for known drugs. Nature.

[8] Kanehisa M, Goto S, Sato Y, Furumichi M, Tanabe M (2012). Kegg for integration and interpretation of large-scale molecular data sets. Nucleic Acids Res.

[9] Knox C, Law V, Jewison T, Liu P, Ly S, Frolkis A, Pon A, Banco K, Mak C, Neveu V, Djoumbou Y, Eisner R, Guo AC, Wishart DS (2011). Drugbank 3.0: a comprehensive resource for ‘omics’ research on drugs. Nucleic Acids Res.

[10] Gaulton A, Bellis LJ, Bento AP, Chambers J, Davies M, Hersey A, Light Y, McGlinchey S, Michalovich D, Al-Lazikani B, Overington JP. Chembl: a large-scale bioactivity database for drug discovery. Nucleic Acids Res. 2011. doi:10.1093/nar/gkr777. 10.1093/nar/gkr777 PMC3245175 21948594

[11] Kuhn M, Szklarczyk D, Pletscher-Frankild S, Blicher TH, von Mering C, Jensen LJ, Bork P (2014). Stitch 4: integration of protein chemical interactions with user data. Nucleic Acids Res.

[12] Jacob L, Vert JP (2008). Protein-ligand interaction prediction: an improved chemogenomics approach. Bioinformatics.

[13] Li H, Gao Z, Kang L, Zhang H, Yang K, Yu K, Luo X, Zhu W, Chen K, Shen J, Wang X, Jiang H (2006). Tarfisdock: a web server for identifying drug targets with docking approach. Nucleic Acids Res.

[14] Xie L, Evangelidis T, Xie L, Bourne PE (2011). Drug discovery using chemical systems biology: Weak inhibition of multiple kinases may contribute to the anti-cancer effect of nelfinavir. PLoS Comput Biol.

[15] Mousavian Z, Masoudi-Nejad A (2014). Drug-target interaction prediction via chemogenomic space: learning-based methods. Expert Opinion Drug Metab Toxicol.

[16] van Laarhoven T, Nabuurs SB, Marchiori E (2011). Gaussian interaction profile kernels for predicting drug–target interaction. Bioinformatics.

[17] Bleakley K, Yamanishi Y (2009). Supervised prediction of drug–target interactions using bipartite local models. Bioinformatics.

[18] Zheng X, Ding H, Mamitsuka H, Zhu S. Collaborative matrix factorization with multiple similarities for predicting drug-target interactions. In: Proceedings of the 19th ACM SIGKDD International Conference on Knowledge Discovery and Data Mining. ACM: 2013. p. 1025–1033, doi:10.1145/2487575.2487670.

[19] Gönen M (2012). Predicting drug–target interactions from chemical and genomic kernels using bayesian matrix factorization. Bioinformatics.

[20] Ezzat A, Zhao P, Wu M, Li X, Kwoh CK (2016). Drug-target interaction prediction with graph regularized matrix factorization. IEEE/ACM Trans Comput Biol Bioinformatics.

[21] Mei JP, Kwoh CK, Yang P, Li XL, Zheng J (2013). Drug-target interaction prediction by learning from local information and neighbors. Bioinformatics.

[22] Chen X, Liu MX, Yan GY (2012). Drug-target interaction prediction by random walk on the heterogeneous network. Mol BioSyst.

[23] Cheng F, Liu C, Jiang J, Lu W, Li W, Liu G, Zhou W, Huang J, Tang Y (2012). Prediction of drug-target interactions and drug repositioning via network-based inference. PLoS Comput Biol.

[24] He Z, Zhang J, Shi XH, Hu LL, Kong X, Cai YD, Chou KC (2010). Predicting drug-target interaction networks based on functional groups and biological features. PloS One.

[25] DRAGON. http://www.talete.mi.it/. Accessed Nov 2016.

[26] Li ZR, Lin HH, Han LY, Jiang L, Chen X, Chen YZ (2006). Profeat: a web server for computing structural and physicochemical features of proteins and peptides from amino acid sequence. Nucleic Acids Res.

[27] Yu H, Chen J, Xu X, Li Y, Zhao H, Fang Y, Li X, Zhou W, Wang W, Wang Y (2012). A systematic prediction of multiple drug-target interactions from chemical, genomic, and pharmacological data. PLoS ONE.

[28] Nanni L, Lumini A, Brahnam S (2014). A set of descriptors for identifying the protein-drug interaction in cellular networking. J Theor Biol.

[29] Xiao X, Min JL, Wang P, Chou KC (2013). igpcr-drug: A web server for predicting interaction between gpcrs and drugs in cellular networking. PLoS ONE.

[30] Cao DS, Liu S, Xu QS, Lu HM, Huang JH, Hu QN, Liang YZ (2012). Large-scale prediction of drug-target interactions using protein sequences and drug topological structures. Analytica Chimica Acta.

[31] Yamanishi Y, Pauwels E, Saigo H, Stoven V (2011). Extracting sets of chemical substructures and protein domains governing drug-target interactions. J Chem Inform Modeling.

[32] He H, Garcia EA (2009). Learning from imbalanced data. IEEE Trans Knowl Data Eng.

[33] Weiss GM (2004). Mining with rarity: A unifying framework. SIGKDD Explor Newsl.

[34] Cao DS, Xiao N, Xu QS, Chen AF (2015). Rcpi: R/bioconductor package to generate various descriptors of proteins, compounds and their interactions. Bioinformatics.

[35] Wassermann AM, Geppert H, Bajorath J (2009). Ligand prediction for orphan targets using support vector machines and various target-ligand kernels is dominated by nearest neighbor effects. J Chem Inform Model.

[36] Breiman L (2001). Random forests. Mach Learn.

[37] Zhou ZH (2012). Ensemble methods: Foundations and algorithms.

[38] Arthur D, Vassilvitskii S (2007). K-means++: The advantages of careful seeding. Proceedings of the Eighteenth Annual ACM-SIAM Symposium on Discrete Algorithms. SODA ’07.

[39] Meir R, Rätsch G, Mendelson S, Smola AJ (2003). An Introduction to Boosting and Leveraging. Advanced Lectures on Machine Learning: Machine Learning Summer School 2002 Canberra, Australia, February 11–22, 2002 Revised Lectures.

[40] Fawcett T (2006). An introduction to roc analysis. Pattern Recognit Lett.

[41] Lim E, Pon A, Djoumbou Y, Knox C, Shrivastava S, Guo AC, Neveu V, Wishart DS (2010). T3db: a comprehensively annotated database of common toxins and their targets. Nucleic Acids Res.

